# PYR/PYL/RCAR Receptors Play a Vital Role in the Abscisic-Acid-Dependent Responses of Plants to External or Internal Stimuli

**DOI:** 10.3390/cells11081352

**Published:** 2022-04-15

**Authors:** Justyna Fidler, Jakub Graska, Marta Gietler, Małgorzata Nykiel, Beata Prabucka, Anna Rybarczyk-Płońska, Ewa Muszyńska, Iwona Morkunas, Mateusz Labudda

**Affiliations:** 1 Department of Biochemistry and Microbiology, Institute of Biology, Warsaw University of Life Sciences-SGGW, Nowoursynowska 159, 02-776 Warsaw, Poland; jakubgraska1@gmail.com (J.G.); marta_gietler@sggw.edu.pl (M.G.); malgorzata_nykiel@sggw.edu.pl (M.N.); beata_prabucka@sggw.edu.pl (B.P.); anna_rybarczyk_plonska@sggw.edu.pl (A.R.-P.); mateusz_labudda@sggw.edu.pl (M.L.); 2 Department of Botany, Institute of Biology, Warsaw University of Life Sciences-SGGW, Nowoursynowska 159, 02-776 Warsaw, Poland; ewa_muszynska@sggw.edu.pl; 3 Department of Plant Physiology, Poznań University of Life Sciences, Wołyńska 35, 60-637 Poznań, Poland; iwona.morkunas@gmail.com

**Keywords:** abiotic stress, ABA receptors, abscisic acid, biotic stress, drought, plant cell signaling, plant defense, phytohormones, protein phosphatases 2C, sucrose non-fermenting 1-related protein kinases 2

## Abstract

Abscisic acid (ABA) is a phytohormone that plays a key role in regulating several developmental processes as well as in response to stressful conditions such as drought. Activation of the ABA signaling cascade allows the induction of an appropriate physiological response. The basic components of the ABA signaling pathway have been recognized and characterized in recent years. Pyrabactin resistance, pyrabactin resistance-like, and the regulatory component of ABA receptors (PYR/PYL/RCAR) are the major components responsible for the regulation of the ABA signaling pathway. Here, we review recent findings concerning the PYR/PYL/RCAR receptor structure, function, and interaction with other components of the ABA signaling pathway as well as the termination mechanism of ABA signals in plant cells. Since ABA is one of the basic elements related to abiotic stress, which is increasingly common in the era of climate changes, understanding the perception and transduction of the signal related to this phytohormone is of paramount importance in further increasing crop tolerance to various stress factors.

## 1. Introduction

Abscisic acid (ABA) is a well-known phytohormone involved in many processes related to plant growth and development, such as seed maturation and germination, flowering, stomatal movement, and senescence, and it mediates the response to adverse abiotic environmental conditions and infestation with pests and pathogens [1,2,3,4,5,6,7]. In plants, the levels of ABA increase not only under the influence of stress factors but also under nonstress conditions due to changes in the intensity of environmental stimuli or in the level of primary metabolites such as sugars [7]. The ABA content in plant tissues is variable and depends on the organ and its development phase. The endogenous concentration of ABA in plant cells is determined by the balance between ABA biosynthesis and catabolism, as well as by the rate of ABA transport to its sites of action [1,4]. Changes in the ABA content in plant tissues trigger the signaling cascade, which depends on the induction of the expression of genes being under the control of ABA. Protein products resulting from the expression of the mentioned genes participate in the development of a response to the optimal physiological conditions or stressors [4].

Changes in endogenous ABA content accompanying the above processes lead to the induction of gene expression, the transcription of which is possible due to the activity of appropriate components of the ABA signaling pathway [8]. Under the conditions of increasing ABA content, the signal is perceived by receptor proteins through ABA binding. Receptor proteins start a signaling cascade, transmitting information via the transmitter to the effector site and developing a response to a stimulus through the resulting products of gene expression under the control of ABA. The functioning of signaling pathways enables precise modification of the genes’ expression and the resulting proteins, leading to the induction of a response to specific conditions of the external or internal plant environment [9].

Key components of the ABA signaling pathway, regulating the sensitivity of tissues to this phytohormone, are receptors from the group of pyrabactin resistance, pyrabactin resistance-like, and the regulatory component of ABA receptors (PYR/PYL/RCAR) binding ABA [10]. In this review, we present, confront, and discuss different recent views on the regulation of the ABA signaling pathway involving PYR/PYL/RCAR receptors in plant cells.

## 2. ABA Signaling Pathway

**Figure 1:**
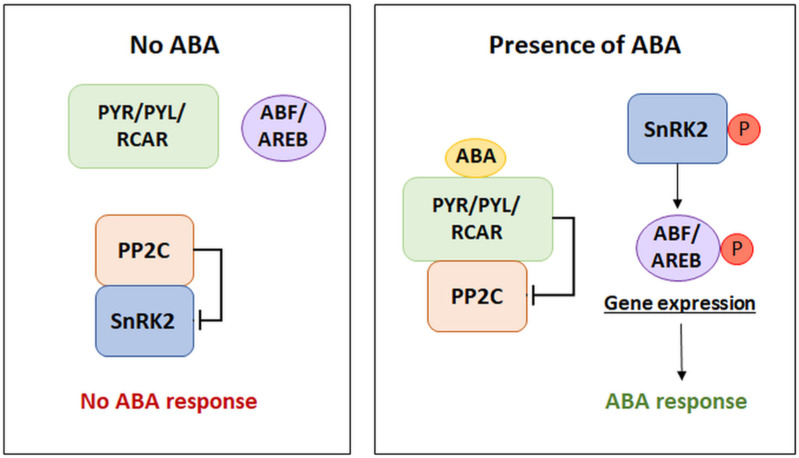
The core components of the ABA signaling pathway. PYR/PYL/RCAR: pyrabactin resistance/pyrabactin resistance-like/regulatory component of ABA receptors, PP2C: type 2C protein phosphatase (negative regulator), SnRK2: sucrose non-fermenting 1 (SNF1)-related protein kinase 2 (positive regulator), ABF/AREB: ABA-responsive element binding factor/protein, P: phosphorylation. (Modified from [12]).

Activation of the ABA signaling pathway occurs due to the recognition of phytohormone molecules by the appropriate receptors, which leads to the induction of the signal transduction cascade. Depending on the tissue and its stage of development, cells contain different amounts of the above receptor proteins. In this signaling pathway, the presence of three main components regulating the perception and transduction mechanism is indicated: PYR/PYL/RCAR receptors, type 2C protein phosphatases (PP2Cs), which are negative regulators of the pathway, and sucrose non-fermenting 1 (SNF1)-related protein kinases 2 (SnRK2s), which are positive regulators of signal transduction [11]. There are various receptor types that are considered to be able to bind ABA. However, the basic mechanism within the interaction of transduction components, in the presence of ABA, is the binding of the phytohormone molecule by receptors from the PYR/PYL group. Consequently, this leads to the blocking of the activity of type 2C protein phosphatases. The lack of PP2C activity causes the activation of SnRK2 protein kinases, which participate in the phosphorylation of the transcription factors—the ABA-responsive element binding factor/protein (ABF/AREB). This mechanism leads to the induction of gene expression under the control of ABA (Figure 1) [12]. The modulation of the activity of individual components is based on the influence of negative regulators of the components of the signal pathway. Such a relationship is observed in the case of the inhibitory effect of type 2C phosphatases on SnRK2 kinases in the absence of ABA availability. By blocking the activity of kinases, PP2Cs prevent the induction of the signaling cascade [13]. Examples of other negative transduction regulators will be discussed in the section on signal transduction termination.

### 2.1. The Dependence of the ABA Signaling Pathway on the Expression of ABA Metabolic Genes

**Figure 2:**
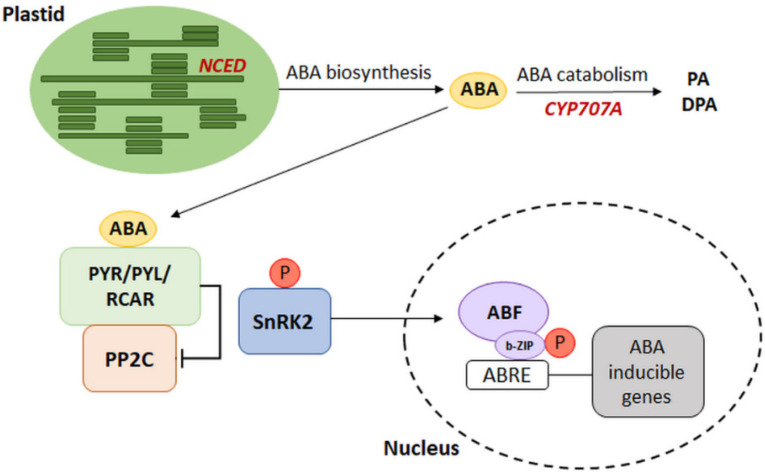
The schematic model of connection between ABA metabolism and transduction pathways. NCED: 9-*cis*-epoxycarotenoid dioxygenase, CYP707A: ABA 8’-hydroxylase, PA: phaseic acid, DPA: dihydro phaseic acid, ABRE: ABA-responsive element, ABF: ABRE binding factor with b-ZIP—leucine zipper domain, PYR/PYL/RCAR: ABA receptors, PP2C: type 2C protein phosphatase (negative regulator), SnRK2: sucrose non-fermenting 1 (SNF1)-related protein kinase 2 (positive regulator), P: phosphorylation. (Modified from [14]).

It is well-known that plants’ acclimatization to changing environmental conditions is often related to changes in ABA metabolism. Responses to ABA-mediated environmental conditions result from fluctuations in the endogenous concentration of phytohormones, which is the result of the equilibrium between its synthesis and catabolism. Understanding of the relationship between metabolism and ABA signal transduction was possible due to the identification of signal pathway components, as well as the identification of genes encoding enzymes involved in ABA biosynthesis and degradation [14]. The most crucial step in ABA synthesis is the conversion of 9-*cis*-violaxanthin and 9-*cis*-neoxanthin into *cis*-xanthoxin (occurring in plastids) by 9-*cis*-epoxycarotenoid dioxygenase (NCED), encoded by the *NCED* gene family. NCED gene expression is a major determinant of ABA accumulation. The synthesis stage catalyzed by NCED enables the formation of the last product of reactions occurring in plastids, i.e., xanthoxin, which is then transported to the cytoplasm where it undergoes further changes leading to the formation of ABA. As a result of biosynthesis, phytohormones may bind to the receptor and initiate the signaling pathway. On the other hand, ABA 8’-hydroxylase (ABA 8’-OH) is the key component involved in ABA catabolism. This enzyme participates in the breakdown of phytohormones by hydroxylation, and it is encoded by the CYP707A gene family [15,16]. Research conducted on *Arabidopsis thaliana* over the last decades have shown significant relationships between genes whose expression accompanies the reactions of metabolism and ABA signal transmission. The simplified model of interaction shows the involvement of several genes related to phytohormone biosynthesis and catabolism, including genes from the NCED and CYP707A families [14] (Figure 2). The presence of ABA initiates the activation of further components of the signaling pathway described above. The attachment of phosphorylated ABF transcription factors to the promoter of a target gene via a leucine zipper domain (b-ZIP transcription factor family) induces the expression of ABA-dependent genes [17] (Figure 2).

### 2.2. Types of ABA Receptors

There are three groups of ABA receptors: the H subunit of the chloroplast magnesium chelatase/ABA receptor (ChlH/ABAR), G protein-coupled receptor-type G protein 1/2 (GTG1/GTG2), and the PYR/PYL/RCAR family, whose representatives are the main signal pathway components capable of binding ABA [12]. ABA receptors are located in various subcellular locations: cytosol/nucleus-localized PYR/PYL/RCARs, chloroplast envelope-localized CHlH/ABAR, and plasma membrane-localized GTG1/GTG2 [18,19].

**Figure 3:**
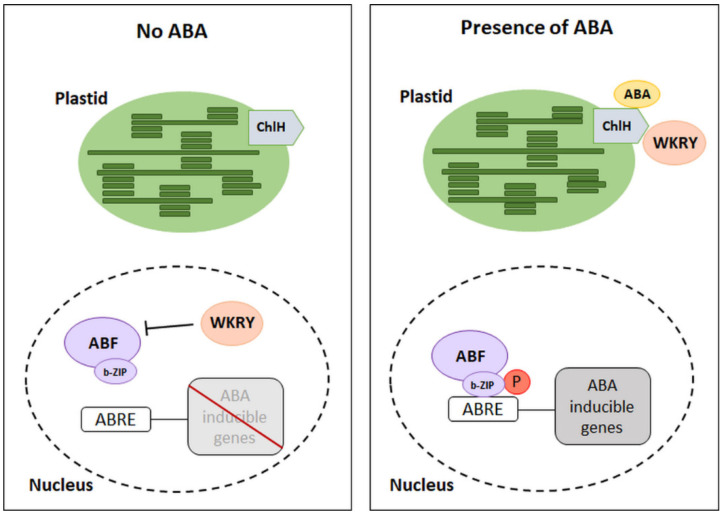
Mechanism of ABA-dependent gene inhibition by WKRY. ChlH: ABA receptors, WKRY: transcription factors (negative regulators of transcription), ABRE: ABA-responsive element, ABF: ABRE binding factor with b-ZIP—leucine zipper domain, P: phosphorylation. (Modified from [14,24]).

ChlH receptors have been identified as ABA binding proteins in *Vicia faba*, and the presence of the homologous ABAR receptor in *A*. *thaliana* [20,21] and *Hordeum vulgare* has been detected, but the ability to bind ABA was not observed in *H. vulgare* [22]. The described receptors can only bind the relevant stereochemical form of the phytohormone, which is (+)-ABA. Its binding to ABA does not directly promote the expression of ABA-dependent genes. In contrast, these proteins are negative regulators of transcription factors that limit the expression of genes dependent on the presence of phytohormones. The C-terminus of the ChlH receptor is in the cytoplasm, it is hypersensitive to ABA, and it is a place of phytohormone molecule binding. Thus, there is an interaction of ChlH with the cytosolic family of WRKY transcription factors (18, 40, and 60). Depending on the content of ABA in the tissues and its binding by receptors, the WKRY factors may inhibit the expression of ABA-dependent genes. Low phytohormone concentration promotes the presence of these factors within gene promoters, thus inducing inhibition of their expression (Figure 3) [19,23]. On the other hand, when the ABA content in the tissues is high, the WRKY factors strongly interact with the ChlH domain, leading to inactivation of the repressive activity of these factors. Thus, the expression of ABA-dependent genes is possible [24]. It has been shown that WRKY factors can function as negative regulators in ABA signaling in seed germination. In this case, the activity of ABA insensitive (ABI5) factor from the b-ZIP family is no longer inhibited [19,23]. However, the exact function of ChlH in other physiological processes is so far not well understood.

The identified GTG receptors, the function of which is related to the G protein, have been located on the surface of the cell membrane in a wide variety of *A*. *thaliana* tissues. GTG1 and GTG2 are believed to have a specific ability to bind ABA. Only in the GTG–GDP state can they bind phytohormone, initiating the signal transduction. GTPase activity is inhibited when GTG is connected to the α subunit of the G protein—GPA1 (G-protein α subunit 1). Under such conditions, the binding of the ABA by the receptors is not possible [25,26]. Using the *A*. *thaliana gtg1*/*gtg2* mutants, a slight sensitivity to ABA during germination was shown. Thus, the participation of ABA in the context of maintaining seed dormancy was almost imperceptible due to the lack of active signaling. Further proteomics-based analysis supported the role of GTG proteins in the regulation of ABA response in Arabidopsis and potentially indicated their possible links with some of the effectors of the ABA signaling pathways [27]. However, a study using independently generated *gtg1/gtg2* mutants suggested a normal ABA sensitivity in seed germination and root growth inhibition [28]. The up-to-date literature does not indicate a significant role of GTG receptors in a wide range of conditions [25].

Among the previously identified ABA receptors, PYR and PYL have the greatest share in ABA binding [19]. Their identification was based on the assessment of the sensitivity of the *A*. *thaliana* mutants to the exogenous application of the ABA analogue pyrabactin. The following groups of receptors have been identified: pyrabactin resistance 1 (PYR1) and other homologues related to PYR1, i.e., PYLs, also known as RCAR receptors [29,30]. These receptors have a conserved steroidogenic acute regulatory-related lipid transfer (START) domain and thus belong to the super family of START proteins. Fourteen genes encoding protein ABA receptors, characterized by a conserved amino acid sequence, have been identified in the Arabidopsis genome. The best-known ABA receptors among monocots (cereals) are rice receptors. Thirteen orthologs of PYL receptors have so far been identified in this species, ten of which are considered active [31]. These proteins can simultaneously bind phytohormone molecules and inhibit PP2C, which leads to the activation of other elements of the ABA signaling pathway [29,30]. Inhibition of the activity of phosphatases of type 2C allows for the restoration of the activity of SnRK2 kinases, the phosphorylation of which leads to the induction of signal transduction and the initiation of the transcription of genes dependent on increasing ABA concentration (e.g., genes related to acclimatization to stress conditions) [32]. Functional analyses of individual PYRs and PYLs indicated the processes in which the activity of these receptors is observed. In the case of the analysis of PYL5, PYL8, and PYL9 in *A*. *thaliana*, an increase in the expression of genes encoding the present receptors was noted under drought [30,33]. In turn, the experiment conducted by Fujii et al. [34] showed that all identified *A*. *thaliana* PYR/PYL receptors (except AtPYL13) can induce signaling due to their receptor activity.

### 2.3. Type 2C Protein Phosphatases—Negative Regulators of ABA Signaling

Reversible phosphorylation is one of the basic mechanisms of regulation of the ABA signal pathway, influencing signal transduction depending on the prevailing environmental conditions and the physiological state of the plant. These reactions are catalyzed by SnRK2 (a transcription factor phosphorylation reaction—signal transduction) and PP2C (a SnRK2 dephosphorylation reaction—signal transduction inhibition), respectively [35]. Seventy-six phosphatases of the PP2C type have been identified in the Arabidopsis genome, of which only Group A is associated with the inhibition of ABA signal transduction. In this group, ABA-insensitive 1 (ABI1), ABI2, ABA-hypersensitive 1 (HAB1), HAB2, ABA-hypersensitive germination 1 (AHG1), and PP2CA/AHG3 are engaged in the negative regulation of the phytohormone signaling pathway. Group A PP2Cs also include highly ABA-induced (HAI1), HAI2, and HAI3, but their role seem to be less important [36,37]. Among PP2Cs, ABI1, ABI2, and HAB1 are the most well-characterized [37].

The contribution of ABI1, ABI2, and HAB1 from all known PP2Cs in the ABA transduction pathway was observed in several experiments aimed at demonstrating the role of PP2Cs in seed dormancy release. Under normal conditions, the low ABA content is not able to inhibit seed germination. A study by Leung et al. [38] showed that Arabidopsis *abi1-1* and *abi2-1* mutants had decreased sensitivity to the ABA inhibition of seed germination and seedling growth. However, analysis of insertion mutants *hab1-1* of the *A*. *thaliana* showed that, after the exogenous application of ABA, the seeds were hypersensitive to phytohormones, and their germination was inhibited [39]. On the other hand, the overexpression of *HAB1* was correlated with a decrease in the seeds’ sensitivity to ABA, thus contributing to their dormancy breaking [39]. It was assumed that proteins encoded by *abi1* and *abi2* genes might act by trapping their endogenous substrates in an inactive complex, while enhanced HAB1 phosphatase activity attenuates the ABA transduction pathway by dephosphorylating positive regulators of ABA signaling. It seems that the target proteins of ABI1 and ABI2 are likely different from those of HAB1 [39].

Moreover, PP2Cs act as negative regulators in ABA-mediated stomatal closure, since they can directly inhibit the guard cell S-type anion channel protein (SLAC1—slow anion channel associated-1). Phosphorylated SLAC1 releases anions from the guard cell and triggers membrane depolarization, leading to the release of potassium ions outwards along with water from the cell, thereby decreasing guard cell turgor pressure and closing the stomatal pore [40,41]. PP2Cs (e.g., ABI1 and PP2CA) can also inhibit SLAC1 through interaction with the open stomata 1 protein (OST1), which is one of the SnRK2 kinases [42,43,44]. Based on the presented examples, these type 2C phosphatases can be considered as key components of negative regulation of the ABA transduction pathway. Additionally, PP2Cs are not only responsible for dephosphorylation of SnRK2, but they also act as negative regulators in some stress mitogen-activated protein (MAP) kinase pathways and in some calcineurin B-like calcium sensors (CBLs)—CBL-interacting protein kinase (CIPK) pathways, as well as in processes involving cyclin-dependent protein kinases [45,46,47].

### 2.4. Interaction of PYR/PYL/RCAR Receptors with PP2C Phosphatases

The selective interaction of PP2C with PYR/PYL/RCAR receptors depends on the current ABA content in the tissues. Thus, it is possible to indicate the functional specialization of ABA receptors in relation to PP2Cs, which guarantees the precise regulation of signal transduction initiation [48]. It is indicated that, of the fourteen identified PYL receptors in Arabidopsis, in the presence of ABA, not all receptors initiate the inhibition of PP2C activity. PYL1, PYL3, PYL6, PYL8, PYL9, PYL10, and PYL11 receptors, after binding phytohormone molecules, inactivated PP2C, thereby initiating signal transduction, thus constituting a positive regulator of the signal transduction pathway [17].

Interactions between ABA and PP2C receptors are extraordinarily complex and still not entirely understood. An analysis of combinations of PYL–PP2C pairs in Arabidopsis by Tischer et al. [49] showed the existence of 126 possible receptor–phosphatase pairs. It was observed that only 113 pairs, when combined, lead to the inhibition of PP2C activity. These studies show the diverse sensitivity of individual PYLs to the presence of ABA, which confirms the current knowledge about the different degrees of affinity of receptors to phytohormones. This experiment also investigated the interaction of PYL with hypersensitive germination 1 (AHG1) PP2C. Unique tyrosine residue in PYL is critical for interaction with AHG1. In addition, in Arabidopsis, all known PYLs showed an interaction with highly ABA-induced PP2C 1 (HAI1) and with another identified HAI phosphatase, HAI3e. A similar interaction between PP2C and tyrosine residue was noticed in transgenic rice, where a conserved region of phosphatase type 2C—VxGPhiL—interacted with the tyrosine residue within the amino acid sequence of the ABA receptor [50].

### 2.5. SnRK2 Kinases as Positive Regulators of the ABA Transduction Pathway

Phosphorylation within the essential components of the signaling pathway is required to initiate ABA signaling. SnRK2 kinases are classified as serine/threonine kinases. Activated SnRK2s are capable of phosphorylating the corresponding transcription factors, ion channels (SLAC1, K^+^ channel in *A. thaliana* 1 (KAT1)), and the plasma membrane-localized respiratory burst oxidase homolog (RBOH) [51,52]. The first ABA-related kinase identified was ABA-activated protein kinase (AAPK) in *V. faba*, acting as a positive regulator of signaling, leading to the stomata closure [53]. The AAPK ortholog in the Arabidopsis, OST1 (or SnRK2.6), has the same function regarding the process of regulation of the stomata opening, as was mentioned above [54]. In addition, several ABA-induced kinases have been identified in *A. thaliana* and rice, the structure and function of which correspond to AAPK of *V. faba* [54]. The significant role of SnRK2s in the activation of genes related to acclimatization to osmotic stress has been indicated [55]. There are three classes of SnRK2 kinases. Kinases belonging to Class I are activated by osmotic stress but not by ABA, those of Class II are weakly regulated by ABA, and those of Class III are strongly activated by ABA [56,57]. Analysis of the *A. thaliana* Class III SnRK2 mutants indicated a key role of this group in ABA signaling, which is induced through serine/threonine residues within the activation loop [34,57]. The identification of SnRK2 explained the mechanisms of activation of the expression of genes whose transcription is induced by the ABA signaling pathway. Moreover, it was possible to reveal the interaction of SnRK2 with phosphatases of type 2C (dephosphorylation of kinases, leading to inactivation of the signaling pathway) and ABA receptors [34,51].

## 3. The Regulation of Gene Expression Dependent on the Presence of ABA

ABA biosynthesis, among others, is a signal for the plant to adjust its metabolism and develop a physiological response to environmental conditions. Under osmotic stress, an increase in the expression of ABA-inducible genes related to acclimatization to unfavorable conditions is observed. There is an interaction of ABF/AREB transcription factors with these gene promoters. By analyzing the promoters of the representative ABA-induced genes, it was found that ABA-responsive gene expression requires multiple *cis*-elements, named ABREs (PyACGTGG/TC) [35,58]. Several of these ABFs, within their amino acid sequence, have a leucine zipper (bZIP) domain and conserved Ser/Thr domains, the presence of which enables the attachment of phosphate residues by previously phosphorylated SnRK2 kinases [59,60]. Studies with the use of *A. thaliana* mutants allowed for the identification of important transcription factors, activated because of the influence of kinases on their protein structure. Factors AREB1, AREB2, and ABF3 were found to be crucial in the regulation of ABRE components in the ABA signaling pathway in response to stress [61]. In addition, studies in subsequent years with the use of *A. thaliana* showed that AREB1, AREB2, ABF3, and ABF1 play significant roles in the ABA signaling pathway during growth [13,62]. This was confirmed by experiments focusing on connecting elements of the ABA transduction pathway, the activity of which leads to ABF phosphorylation and thus to their activation. It has been pointed out that, among the previously identified SnRK2s in Arabidopsis, Subclass III kinases play a key role in this process. The cellular localization of this SnRK2 subclass revealed their presence in the cell nucleus, which is analogous to the AREB/ABF factors (Figure 2) [55,58].

SnRK2 kinases, through their activity in several processes associated with the ABA, can lead to the activation of transcription factors. In addition to this, the family of ABI5 transcription factors has been identified. The presence of ABI5 has been observed in both seed germination and dehydration stress. In the presence of ABA, Class III SnRK2 kinases catalyze the phosphorylation of ABI5 to regulate ABRE, which leads to promotion of the expression of ABA-dependent genes [63]. Research aimed at identifying the nature of ABI5 degradation emphasized the special role of other transcription factors from the bZIP family (ABI3 and other ABFs) in the regulation of the expression of genes related to cell dehydration [63,64].

## 4. Structure, Characteristics, and Functioning of PYR/PYL Receptors

**Figure 4:**
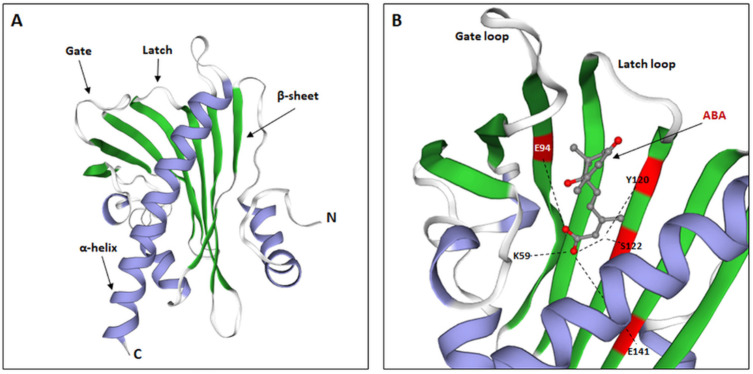
ABA receptor structure. (**A**). Structure of the monomeric, apo-form of *Festuca elata* PYR1 (FePYR1), showing a secondary structure: α-helixes, β-sheets, and two main loops, involved in the ABA binding: latch and gate. (**B**). Structure of AtPYR1 binding pocket, highlighting main amino acids involved in the interaction with ABA residues: K59, E94, Y120, S122, and E141. (Modified from [71]).

Studies of the crystallographic structures of the *A. thaliana* PYR/PYL receptors in a non-ABA form have confirmed the presence of the amino acid START domain within their structure, which is characteristic of the START superfamily [29,30]. Due to their similar structure, these proteins are classified together with the Bet v 1-fold superfamily of proteins (the family name comes from the allergen contained in *Betula verrucosa* pollen) [65,66]. It is indicated that PYL receptors contain the helix grip structure, which is characteristic of the above-mentioned protein families [33,67,68,69,70]. Based on the newest conducted bioinformatics analysis, its structure comprises a seven-stranded β-sheet, flanked by two α-helices, which is designated as a helix-grip fold. Additionally, the PYR/PYL family contains a α-helical segment at the N-terminus (Figure 4A). The organization of individual receptor elements determines the formation of a hydrophobic pocket capable of binding the ligand, i.e., ABA [71].

Elements that are essential for the proper function of the binding pocket are the helical sections inside the polypeptide, present, inter alia, between closely located β-sheets. They form loops with a conserved amino acid sequence, capable of ABA binding and initiation of the signaling cascade [66,67]. The most important sections are the gate loop between β3 and β4 (preserved GLPA amino acid sequence) and the latch loop between β6 and β7 (preserved HRL amino acid sequence) [72]. Thus, the correct receptor pocket is present between the α-helix of the C-terminus of the protein and the loops. These loops remain bent in their non-ABA state, which keeps the receptor pocket open and in contact with the environment. Conformational changes leading to ABA binding are possible only when the phytohormone is in the appropriate stereochemical form. As shown by the study of Park et al. [29], the preferred enantiomer of phytohormone is the (+)-ABA form. In contrast, it is indicated that the (-)-ABA form can also bind to PYL receptors, but with a lower affinity. Moreover, studies on the crystallographic structures of the receptors revealed that PYL proteins bind to ABA molecules with a molar ratio of 1:1.

### 4.1. Structure of the Internal Pocket of PYL Receptors and Their Ability to Bind ABA

In the research on *A. thaliana* mutants, it was observed that the internal structure of the ABA binding pocket in the identified PYL receptors closely corresponds to the functional groups present in the chemical structure of the phytohormone [33,67,68,69,70]. ABA is a sesquiterpene, so it has groups with hydrophobic properties. The cyclohexene ring and the isoprene unit, present in the phytohormone structure, together with the side chains of PYL receptors, create non-polar hydrophobic interactions. Moreover, it was observed that the polar side chains of the spatial structure of PYL receptors form hydrogen bonds with numerous groups of ABA molecules—hydroxyl, carboxyl, and ketone groups. Based on the analysis of the amino acid sequence of the Arabidopsis PYR1 receptor, the identification of the amino acids involved in the interaction with ABA residues was performed. Hydrogen bonds are believed to form between the carboxyl group of the ABA molecule and the glutamic acid residues at Positions 94 and 141 (E94, E141), tyrosine 120 (Y120), and serine 122 (S122) (Figure 4B). Meanwhile, lysine at Position 59 (K59) is recognized as the key amino acid in the structure of the receptor, responsible for interactions with the polar chains of phytohormone (Figure 4B) [33,73]. It has been found that a mutation within this amino acid position renders the receptor unable to bind to ABA, and the inhibition of PP2Cs, required for further activation of the components of the ABA signaling pathway, is inactivated.

### 4.2. The Degree of Oligomerization of the Structure of PYL Receptors

Hao et al. [74] systematized the knowledge about the various biochemical properties of the *A. thaliana* PYR/PYL receptors identified in 2009 by numerous teams [33,67,68,69,70]. The conducted experiment demonstrated that the receptors differ in the degree of oligomerization, which influences the way in which PYL/PYR receptors inhibit PP2C activity. This process can take place with or without the presence of ABA. It has been shown that AtPYR1 and AtPYL1-2 exist only as homodimers and require ABA binding to inhibit PP2Cs. Conversely, it has been observed that the monomeric form of AtPYL10 is able to inhibit PP2C independently of ABA binding [74].

The identification and characterization of PYL receptors in rice by He et al. [75] confirmed a similar phenomenon as described for *A. thaliana*. Among the 12 identified OsPYL rice proteins, the group of receptors that formed the dimer structure required the presence of ABA to inhibit the activity of type 2C phosphatase, as opposed to the monomeric forms. It was noticed that monomeric OsPYL12 also has strong inhibitory properties. This protein was able to inhibit the two forms of the identified PP2C in rice, independently of ABA binding [75]. Bioinformatic analysis revealed the similarity of the amino acid sequence of OsPYL12 (Q38 and F71) and AtPYL13 (Q48 and F76), which confirmed the close evolutionary relationship of both proteins [76]. Isothermal titration calorimetry studies have shown that OsPYL12 is unable to bind ABA. Thus, it was confirmed that both indicated proteins are orthologs (AtPYL13 can inhibit one of the tested OsPP2Cs) [75]. Moreover, the close relationship of the *A. thaliana* receptors and rice in terms of the oligomerization of the structure was proved by phylogenetic analysis.

### 4.3. Conformational Changes in the PYL Receptor as an Effect of ABA Binding

**Figure 5:**
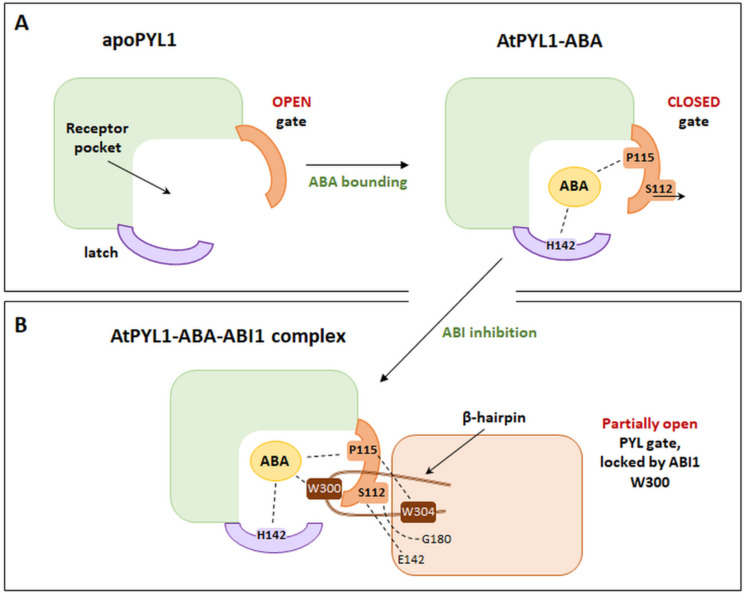
Schematic representations: (**A**). The AtPYL1-ABA complex, highlighting main amino acids involved in the interaction with ABA: S112, P115, and H142. (**B**). The AtPYL1-ABA-ABI1 complex, illustrating the most important amino acid positions of ABI1, involved in the interaction with AtPYL1-ABA (W300, W304, E142, and G180). (Modified from [77]).

Binding of ABA in the binding pocket of the PYL receptor is induced by conformational changes in the protein structure. The binding of the phytohormone by the receptor occurs, among others, by creating a hydrogen bond between the ABA carboxyl group and the conserved sequence of the protein loop [77]. The presence of ABA induces structural changes in the gating and latching loops (Figure 5A). A proline residue (P115 for AtPYL1) present within the gating sequence shifts towards the ABA cyclohexene ring. The serine at Position 112 (S112 for AtPYL), by “bending” the indicated loop, is removed from outside of the protein, which allows the ABA to be bound inside the binding pocket [67,68]. Moreover, because of conformational changes, the histidine residue (H142 for AtPYL1) of the latch loop enters the receptor pocket and interacts with the ABA, thereby contributing to its binding. It is significant in inhibiting the activity of type 2C phosphatases. Only the creation of the closed structure of the PYL receptor can lead to the increased affinity for ABA, the inhibition of the indicated phosphatases activity, and the activation of signal transduction [29,30].

### 4.4. PYL Receptors as Essential Elements of the Plant Stress Response: Lessons from the Study of Drought

Drought is one of the main factors disturbing the physiological balance of plants, leading to lower plant production efficiency worldwide [78]. As a result of water imbalance in a plant caused by a water deficit in the soil, compatible solutes are accumulated in the tissues [79]. Moreover, there is an induction of signaling pathways, leading to the development of a stress response [4,80]. In many abiotic stresses, such as drought, salinity, or cold, the activation of many molecular mechanisms depends on the ABA content in the tissues [81,82]. Endogenous changes in ABA concentration enable the initiation of a signaling cascade, the activation of which depends on the binding of the ABA by appropriate receptor proteins. PYR/PYL/RCAR receptors are believed to be the main component responsible for the regulation of the ABA signaling pathway during plant response to environmental stresses. As a result of signal transduction, the expression of ABA-dependent genes is initiated, so a response to drought, for example, is developed [82,83,84].

Expressions of different genes encoding PYR/PYL/RCAR vary throughout development and in response to stress conditions. Moreover, different expression patterns of these genes have been observed in various tissues. Literature examples of several identified genes encoding PYL receptors in monocotyledons indicate the diverse role of individual genes in the response to abiotic stress, including drought. Functional analysis of the Arabidopsis transgenic plants with overexpression of maize PYL-encoding genes (*ZmPYL3*, *ZmPYL8*, *ZmPYL9*, *ZmPYL10*, and *ZmPYL12*) showed a key role for *ZmPYL8* and *ZmPYL12* in the development of responses to soil water deficits [81]. Moreover, it was observed that these genes contribute to the natural resistance of maize to drought. A study by Fan et al. [85], using 15-day-old maize seedlings treated with polyethylene glycol (PEG), showed an increase in the mRNA level of the *ZmPYL1*–*ZmPYL11* genes in comparison to non-treated seedlings. A significant increase in the level of the transcript was observed with *ZmPYL4*, *ZmPYL7*, and *ZmPYL8*. Moreover, overexpression of the rice *OsPYL9* gene in Arabidopsis resulted in a higher resistance of these plants to drought, compared to wild-type (WT) plants [86]. In turn, the analysis of the transgenic lines of rice overexpressing *OsPYL9* conducted by Usman et al. [87] confirmed the trend previously described by Zhao et al. [86]. Similar results were obtained for Arabidopsis plants with an overexpression of rice *OsPYL3* [88]. However, in sorghum plants under drought stress, most genes encoding SbPYLs were downregulated. Only the expression of *SbPYL1* and *SbPYL7* was almost two-fold higher in leaves under drought treatment compared to untreated controls. In the roots of sorghum plants, all studied SbPYLs genes were downregulated under drought [89].

Furthermore, in dicotyledons, a different involvement of individual PYL receptors in response to drought is noticed. An expression analysis of PYL-encoding genes with the use of microarrays and quantitative reverse transcriptase-PCR in 6–7-week-old dehydrated tobacco plants showed groups characterized by a different transcript profile under the drought conditions [90]. It was concluded that the *NtPYL19–27*, *NtPYL28*, and *NtPYL29* genes did not participate in the tobacco drought response due to the low level of mRNA during stress. On the other hand, among tobacco genes, *NtPYL1–5*, *8*, *9*, *13*, and *14* took part in the discussed responses. These genes were characterized by a constant level of mRNA throughout the experiment. The group consisting of *NtPYL7*, *10–12*, and *17* showed an increase in expression in the initial hours of dehydration (0.5 and 1 h) and a decrease in the following hours (2, 4, and 8 h). In addition, it was recognized that *NtPYL6*, *15*, *16*, and *18* were characterized by a constant level of the transcript in the first hours of drought, and the amount of mRNA was then significantly reduced [90]. Diverse expression patterns of *NtPYLs* suggested not only the participation of some PYL-encoding genes in the drought response, but also indicated that different genes participate in early (*NtPYL7*, *10–12*, and *17*) and late stress response (*NtPYL6*, *15*, *16*, and *18*).

In grape cuttings, the expression of *VvRCAR3* and *VvRCAR7* was induced in leaves under drought. However, other genes encoding receptors were not upregulated in response to drought or were even downregulated (*VvRCAR1* and *VvRCAR5*). In the roots of grape cuttings under drought, all studied *VvRCARs* were downregulated, with the exception of *VvRCAR4* [91].

Analysis of Arabidopsis transgenic plants with overexpression of the *PYL8*/*RCAR3* (*PYL8ox*) gene and with the silenced *PYL8i* gene demonstrated the role of *AtPYL8* in drought tolerance development [92]. Under field conditions, it was observed that, after two weeks of non-irrigation, *PYL8ox* plants showed slight drought-related damages. On the other hand, in *PYL8i* and WT, a strong response to stress was noticed (growth inhibition and wilting). Moreover, in the discussed study by Lee et al. [92], an analysis of the expression of 14 identified genes encoding AtPYL receptors in *PYL8ox*, *PYL8i*, and WT, treated with ABA, was performed to assess the sensitivity of plants to ABA. All the other 13 PYL-encoding genes except *AtPYL8* were shown to have a similar transcript level profile, regardless of whether it was assayed in WT plants or transgenic plants. Thus, the authors concluded that the elevated level of mRNA in *PYL8ox* and WT plants proves that *AtPYL8* participated in the induction of the signaling pathway in drought conditions, shaping the resistance to this stress [92]. This might be due to the adjustment of stomatal aperture under a water deficit. Results by Gonzalez-Guzman et al. [93] for Arabidopsis with different combinations of *pyr*/*pyl* mutations showed that PYR/PYL receptors play a major role in ABA signaling required for the regulation of stomatal conductance. In these mutants, the steady state of stomatal conductance of whole Arabidopsis rosettes was increased compared to the wild type plants, and in sextuple *pyr*/*pyl* mutants, stomata did not close even under ABA treatment.

### 4.5. Model of PYL Receptor–ABA–PP2C Interaction

Crystallographic analysis revealed the mechanism of the formation of a complex consisting of AtPYL1, an ABA bound to the binding pocket and inactivated type 2C phosphatase (ABI1) [67,68,70]. As observed, the sites of interaction of the receptor and phosphatase are the loops in the PYL structure closing the binding pocket, the C-terminus of the receptor helix, and the β-hairpin structures of phosphatase. Based on these studies, tryptophan at Position 300 (W300) and arginine at Position 304 (R304) are the most important amino acid positions within the β-hairpin for ABI1 (Figure 5B). Conformational changes in PP2Cs lead to the insertion of the W300 side chain between the receptor loops (gate and latch). Thus, W300 interacted with the carbon at the 4-position (C4′) of the ABA molecule previously bound in the receptor pocket [70,82,94]. It is believed that the amino acid position within phosphatase is a key element of the complex, which influences the sensitivity of Arabidopsis plants to ABA [94]. An experiment by Takeuchi et al. [95] confirmed the role of conserved tryptophan within the amino acid sequence of type 2C phosphatase. The study was based on the analysis of the crystallographic structures of the PYL–ABA–PP2C complex, but the receptor was bound to modified ABA analogues. The so-called novel analogue of ABA (novel PYL antagonists, PAN) was a 4’-O-phenylpropynyl ABA, modified in the C4′ position of phytohormone. It was shown that this modification at the 4′ position caused a block of PYL–PP2C interactions. This was due to the obstruction of the insertion of the PP2C tryptophan into the tunnel adjacent to the C4′ of ABA, which is crucial for the formation and stabilization of the ABA–PYL–PP2C complex. In vitro PAN caused the abolishment of the ABA-induced PYL–PP2C interactions, and in vivo, it suppressed stress-induced ABA responses in Arabidopsis.

Another essential site in the amino acid sequence of ABI1 contacting PYL1 is arginine at Position 304 (R304). It is believed that this amino acid affects the attraction of the gating loop by interaction with the proline P115 of PYL1. Such a conformation of the complex determines that the active site of PP2Cs is surrounded by the bent structure of the receptor loop (Figure 5A,B) [68,77]. The hydrogen bonding between the serine of the PYL1 gating loop (S112) and the PP2C glutamic acid residue (E142) is also observed in this complex [68]. The S112 of the PYL1 receptor was also shown to interact with glycine at Position 180 in ABI1 (G180). Based on the analysis of the *abi1-1* mutants of Arabidopsis, it was concluded that, within the ABI1 phosphatase, a mutation of a missense character (conversion of glycine 180 into aspartic acid) may occur. It is assumed that this mutant protein is not regulated by ABA, and it shows constitutive activity, which is sufficient to inhibit ABA responsiveness. This occurs because the interaction between the receptor’s gate loop and the active center of phosphatase is hindered [67,96,97,98].

### 4.6. Model of the PP2C–SnRK2 Interaction

**Figure 6:**
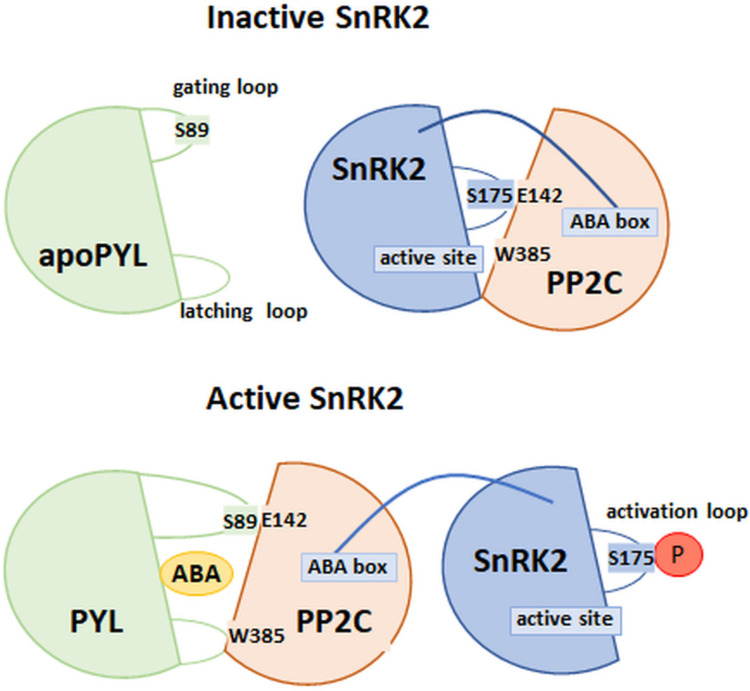
Model of interaction between SnRK2 and PP2C in the absence and presence of ABA. Abbreviations include apoPYL/PYL: ABA receptor (not bound/bound with ABA) with highlighted gate (S89) and latch loops, SnRK2: kinase with highlighted important amino acid position in activation loop (S175: phosphorylation site) and ABA box (interaction with PP2C), PP2C: phosphatase with highlighted W385 and E142 amino acids, as an essential element for activation/inhibition of SnRK2, P: phosphorylation. (Modified from [99]).

The interaction between PP2C and SnRK2 was determined by the analysis of the crystallographic structures of phosphatase–kinase complexes in the absence and presence of ABA (Figure 6). The work of Soon et al. [99] systematized the knowledge about the interaction between the above components of the ABA signaling pathway. The researchers looked at examples of structures formed between HAB1 phosphatase and SnRK2 kinase. It is believed that the sites of interaction between these enzymes include an SnRK2 activation loop connecting to the active site of HAB1 and a tryptophan residue with a conserved position in the phosphatase amino acid sequence. ABA bound in the receptor pocket interacts with tryptophan, which is a conserved amino acid position in the β hairpin structure (W385 in HAB1). During the period of unavailability of ABA, phosphatase interacts with kinase. In the absence of ABA, the W385 of HAB1 interacted with the SnRK2 kinase active site. In conclusion, the mechanism of interaction of the components of the PYL–ABA–PP2C and PP2C–SnRK2 complexes is similar [77,99].

The second site of interaction of HAB1 and SnRK2 described by Soon et al. [99] is the so-called ABA box—the region in the kinase domain composed mostly of acidic amino acids. This structure interacts with the positively charged surface area of HAB1, which, in the absence of the receptor binding of ABA, leads to the inhibition of SnRK2 phosphorylation (Figure 6). Thus, the ABA signaling pathway is not activated [35,99,100]. Studies of the *A. thaliana snrk2.6* mutants, with deletions in the ABA cassette coding sequence, showed no in vitro signs of a loss of kinase function. On the other hand, in in vivo conditions, the process of closing the stomata in these mutants was disturbed, which would indicate that the activation of further components of the ABA signaling pathway depends on the phosphorylation of amino acid positions within this SnRK2 domain [54,100]. This was confirmed by the data obtained by Righetto et al. [101], using recombinant stress-activated SnRK2 regulatory domains (ABA-activated protein kinases; SAPK) isolated from sugar cane. It was shown that, in ScSAPK8, deletion of the ABA cassette led to a reduction in the number of autophosphorylated sites in the protein. In addition, it was observed that the phosphorylation of major regulatory sites in the SnRK2 amino acid sequence (conserved serine residues in the structure, within the P loop and the activation loop) may be sufficient for the full activity of kinases, thus leading to their phosphorylation and initiation of expression of genes, which are dependent on the ABA signal pathway.

According to the most recent findings, the inhibition of SnRK2 by type 2C phosphatases most likely occurs in two stages [99,102]. In the first step, phosphatase (ABI1, ABI2, or HAB1) leads to dephosphorylation of the conserved serine residue in the activation loop (at Position 175 for SnRK2.6). Phosphorylation of this amino acid position is required for full enzyme activity. It should be emphasized that the enzymatic activity of SnRK2 in this step is not completely inhibited. This can occur with a low content of PP2C molecules in relation to SnRK2. In the next stage, the kinase is completely inhibited by creating interactions between the previously described sites of interaction of both enzymes. In this way, a stable structure of the PP2C–SnRK2 complex is created, the form of which may be disturbed only after the ABA is bound by the PYL receptor.

### 4.7. Degradation Pathway for ABA Receptors

The available literature data do not clearly show how the degradation of PYL receptors occurs. It has been observed that the regulation of the level of receptor proteins capable of binding ABA is a unique way of adapting plants to their current physiological state and the prevailing environmental conditions [103]. It has been indicated that the elements of an endosomal sorting complex required for transport (ESCRT) participate in the ubiquitination of ABA receptors in Arabidopsis (PYL 4, 5, 8, and 9) [104,105]. ESCRT consists of the FYVE1 and VPS23A proteins that sort receptors for vacuolar degradation, and of ALG-2-interacting protein X (ALIX), which directly interacts with endosomal receptors, promoting their proteolysis. In turn, research by Zhao et al. [106] showed that, in Arabidopsis plants with overexpression of *PYL9*, subjected to dehydration, the regulation of the content of receptor proteins was conducted through the ubiquitination pathway, with the participation of homologous E3 ligases: PUB22 and PUB23.

## 5. ABA Signal Termination by Inhibitors

Modulation of the activity of individual components of the ABA signaling pathway is based on the influence of negative regulators on these components. The action of these factors may influence the change in the intensity of the response to the stimulus or lead to the termination of the signal transmission. Recent literature data indicate a vital role of negative regulators in modulating the stability of components of the transduction pathway, as well as their influence on tissue desensitization on the stimulus, i.e., ABA. Desensitization occurs because of changes in the content of phytohormones, the reduction of which, in tissues, induces the inhibition of signal transmission and the expression of genes under the control of ABA [103,107,108].

An essential element of the post-translational control of protein stability and activity is the ubiquitin proteasome system (UPS). The present complex participates in protein labeling in the ubiquitination process. UPS combines the activity of three different enzymes: E1 activates the ubiquitin molecule using ATP, E2 is a conjugating enzyme that creates a thioester bond between its structure and ubiquitin, and E3 ligases bind ubiquitin to the protein to be degraded. It has been indicated that the specificity of the proteins degraded throughout the system depends on the E3 ligases [103,107]. Moreover, this type of modulation regulates various elements of phytohormone signaling pathways, including ABA, at all steps [108]. One of the types of E3 ligases is the really interesting new gene (RING), which deserves special attention. Several identified E3 ligases of the RING type in Arabidopsis regulate cellular processes through their activity, including ABA and auxin signaling. They also participate in the germination, in the early stage of seedling growth, as well as in acclimatization to drought [109].

## 6. SnRK2 Degradation with Inhibitors

Activation of ABA-controlled genes strictly depends on both the phosphorylation status of SnRK2 kinases and the activation of transcription factors interacting with them. Dephosphorylation and inactivation of SnRK2 occur not only through the inhibitory effect of PP2C, but also through the ubiquitination cascade [103]. E3 ligases are particularly involved in the degradation of SnRK2 during signal termination, with decreasing stimulus intensity. It is worth mentioning the high osmotic stress gene expression 15 (HOS15) protein, which is a receptor for an E3 ligase-linked ubiquitin molecule of the CULLIN4 type to the SnRK2.6 (OST1) kinase in Arabidopsis [110,111]. HOS15 was observed to mediate cold-induced degradation of the histone deacetylase 2C, acting within the promoter of the cold-responsive (COR) gene. Increased histone acetylation caused a change in the chromatin structure, leading to its relaxation, thus inducing the activity of C-repeat binding transcription factors (CBF) within the COR gene—promoting the production of a stress response [110,111]. The experiment of Ali et al. [111] showed that HOS15 interacts with the OST1 kinase and with ABI1 and ABI2 phosphatases. At the same time, it was observed that this protein plays the role of a negative regulator only in relation to OST1. Researchers observed that *hos15-2* mutants, with the silenced *HOS15* gene, in contrast to WT plants had accumulated and held a stable level of OST1 protein. On the other hand, *hos15-2* mutants were characterized by a significant hypersensitivity to ABA during germination while increasing drought tolerance [111]. It was indicated that, with the increased accumulation of kinases and the lack of a factor conducive to their degradation, signal transduction and responses to stress were induced. Moreover, it was detected that the dehydrated *hos15-2* mutants strongly induced the expression of ABA-controlled genes and genes whose expression depends on cell dehydration.

### Model of HOS15 Participation in the ABA Signaling Pathway

**Figure 7:**
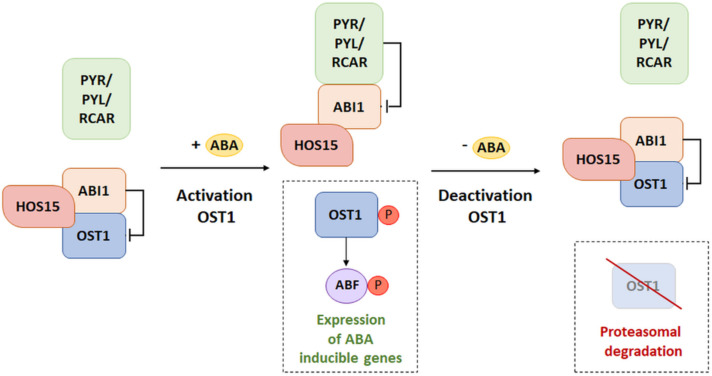
Inhibitory effect of HOS15-ABI1 on the ABA transduction pathway by degradation of OST1. PYR/PYL/RCAR: ABA receptors, ABI1: type 2C protein phosphatase (negative regulator), OST1: SnRK2 kinase (positive regulator), HOS15: receptor for an E3 ligase-linked ubiquitin molecule, ABF: ABRE binding factor, P: phosphorylation. (Modified from [103]).

The model of HOS15 influence on signal transduction assumes that, if the ABA is not bound by PYR/RCAR receptors, ABI1/2 phosphatase dephosphorylates the OST1 kinase, thus leading to inhibition of its activity (Figure 7) [111]. On the other hand, in a situation where ABA is available and bound by PYR, the receptors interacting with the ABI structure inhibit their activity. This state determines the autophosphorylation of OST1 and the phosphorylation of transcription factors (ABI5/ABFs) of genes whose expression depends on ABA. Thus, it is presumed that the presence of ABA weakens the interaction between HOS15 and OST1. Literature data do not clearly define the association of the presence or absence of ABA and the interaction of HOS15 with ABI1/2. In the presence of ABA, activation of the transduction pathway by the release of OST1 from the HOS15-ABI1/2 complex was observed. On the other hand, during signal termination, with decreasing sensitivity of tissues to ABA, interaction of this complex with OST1 was observed, which led to kinase degradation. The studies conducted so far indicated that, during the long-term ABA stimulus both de novo OST1 biosynthesis activation and the accumulation of ABI1/2 phosphatases were observed. This sequence of events promotes the dephosphorylation of OST1 kinases. Unphosphorylated kinases are preferred as a substrate by HOS15. Thus, their degradation may occur through the ubiquitin–proteasome system. The model of the ABA signaling pathway with participation of HOS15 proteins shows the complex process of the inhibition of individual elements of the signal transduction pathway. The presented data suggest that the role of HOS15 is based not only on the inhibition of the ABA signaling pathway, but also on the change in the intensity and duration of signal transmission [103].

## 7. Conclusions

In recent years, the basic components of the ABA signaling pathway have been recognized and characterized. Unfortunately, the current data do not clearly indicate all the functions performed by ABA signal transduction components, under optimal environmental conditions or under stresses. This is because the elements involved in ABA signal transduction have so far not been analyzed in detail in many plant species. Thus, it is postulated that understanding the role of these components, including PYL receptors, is particularly important for more complete insights into the mechanisms of regulation of the ABA signaling cascade during normal plant growth and development, as well as the response to numerous stresses, the frequency and intensity of which become increasingly severe and noticeable on a global scale. The ABA signaling enables an adequate response to stress, in particular to abiotic stress, in plants; however, triggering the response is possible due to the stimulus received by the receptor, hence the key role of PYL/PYR/RCAR receptors. Changes in the number and sensitivity of ABA receptors in various plant organs may be of key importance in the sensitivity of the entire plant to specific stressors, such as drought or salinity. However, it should be remembered that ABA can be a negative stress tolerance regulator, especially with respect to biotic stress. In the presence of both biotic and abiotic stresses, a strong ABA response may increase the plant’s susceptibility to pests/pathogens. Therefore, biotechnology may be useful for changing the sensitivity of a plant to ABA, and this may lead to the creation of varieties that have more balanced responses to multistress and thus that are better adapted to a changing environment.

## Data Availability

Not applicable.
